# Society Meetings

**Published:** 1917-11

**Authors:** 


### SOCIETY MEETINGS

**The Federated Alumni of the University of Buffalo,** at a meeting last month, elected the following officers: Pres., Loran L. Lewis, Pres. of the alumni of the Law Dept.; Vice-Pres., Dr. Wm. G. Bissell, Pres. of the alumni of the Medical Dept.; Sec., Dr. G. Bertram Lemon of the Pharmacal alumni; Treas., Dr. J. G. Roberts of the Dental alumni. Local Alumni Associations will meet as follows: Chautauqua Assn., at Salamanca Nov. 15; Central and Northern N. Y. Assn., at Syracuse, Jan. 15; Metropolitan Assn. in N. Y. City, Jan. 16; Interstate Assn., at Elmira, Mch. 25; Rochester District Assn., at Rochester, Apr. 18. The annual meeting of the Federated Alumni will take place in Buffalo, on University night, Feb. 22.

**The Wyoming Co. Medical Society** at its meeting of Oct. 14 elected officers as follows: Pres., J. E. Slaught, Warsaw; Vice-Pres., L. E. Stage, Bliss; Sec.-Treas., L. M. Andrews, Warsaw; Censors, M. J. Wilson, Warsaw; P. S. Goodwin, Perry; W. J. French, Cowlesville; Delegate to State Society, L. H. Humphrey, Silver Springs; Alternate, J. B. Browlness, Perry.

**The Genesee Co. Medical Society** held its annual meeting at Batavia, Oct. 4. Officers were elected as follows: Pres., R. M. Andrews, Bergen; Vice-Pres., E. F. Will, Batavia; Sec.-Treas., Edith F. Ryan, Batavia.

**Elmira Academy of Medicine.** At a regular meeting, Oct. 3, the following program was presented: Glands of Internal Secretion in Relation to Eye, Ear and Throat, B. G. Voorhees, Uterine Fibromata, R. G. Loop, title not stated, H. E. Batten of Corning. Pres. R. B. Howland, Sec. O. J. Bowman.

**The Medical Society of the Co. of Monroe** held a special meeting Oct. 9, in connection with the Auxiliary Medical Defense Committee of the County, at which Major Henry D.

Jump, M. R. C., showed moving pictures and spoke of the need of surgeons for the war.

---

**Rochester Academy of Medicine,** Section II met Oct. 10, various members presenting unusual cases and specimens. Wm. I. Dean, Chairman, L. W. Jones, Sec.

---

**The American Laryngologic Assn.** has sent us abstracts of its 39th annual congress. We have not the space to publish them but any one interested may consult them at this office.

---

**The Buffalo Academy of Medicine** opened the season with the meeting of the Section of Surgery at Orpheus Hall, 432 Franklin St., Oct. 3. (Meetings will be held regularly at this hall Wednesday evenings at 8:30 and visiting physicians are cordially invited. They should present themselves to some member and secure the privilege of the floor for discussion). Dr. Hugh A. Auchincloss of N. Y. gave a paper on Significance of Clinical Signs of Malignancy in Diagnosis of Conditions of the Mammary Gland. Jas. B. Cross, Chairman, J. F. Brennan, Sec.

The Section of Medicine met Oct. 10. Dr. Elliott P. Joslin of Boston spoke on New Ideas of Diabetes. Chairman, DeWitt H. Sherman, Sec. Jacob S. Otto.

The Section of Obstetrics and Gynaecology met Oct. 17. Dr. H. E. Hayd read a paper on The Thyroid in Gynaecology, Dr. N. Kavinoky on Conservative Treatment of Pelvic Infection. H. K. DeGroat, Chairman, Edward Meister, Sec.

---

**The Medical Society of the County of Erie** held its regular meeting in the Buffalo Medical College on October 8th, 1917. In the absence of President Potter, Vice-President Dr. George F. Cott presided. The minutes of the previous meeting and of the meetings of the Council were read and approved.

The following were elected to membership: Drs. Myron A. Thompson, Robert P. Dobbie, Earl L. Eaton, William H. Jones, Lyman J. Strong, Gertrude Davies, Harold J. Reist, Howard D. Hoehman, Hiram S. Yellen, Vincent C. Muscato, Rosco De Dominicis. Dr. Bernhard H. Brady was reinstated.

The following ticket was nominated for the annual meeting in December: For Pres., Dr. George F. Cott; 1st Vice-Pres., Dr. James E. King; 2d Vice-Pres., Dr. Earl Lothrop; Sec., Dr. Franklin C. Gram; Treas., Dr. Albert T. Lytle. Board of Censors, Drs. John D. Bonnar, Francis E. Fronczak, A. G. Bennett, A. D. Carpenter, F. A. Valente. Chairmen, Committee on Legislation, Dr. Harvey R. Gaylord; Committee on Public Health, Dr. Nelson G. Russell; Committee on Membership, Dr. William F. Jacobs; Committee on Economics, Dr. John V. Woodruff.

Major Henry D. Jump of the Surgeon-General's office addressed the Society, and showed several reels of war pictures. The Major's address met with a hearty vote of thanks by the Society.

FRANKLIN C. GRAM, M. D., Secretary.

---

The regular meeting of the **Gross Medical Club** was held at the Roycroft Inn, East Aurora, Friday, October 19th, being entertained by Dr. W. H. Phelps. Dr. Jas. E. King, presided.

On motion of Dr. J. Henry Dowd the following men, members of the club, but now in the U. S. Service were continued on the active list, their dues and entertainments to be eliminated until after their return: Majors Wende, Clinton and Russell; Capts. Cott and McKenny.

Dr. A. G. Bennett read a very interesting paper, on "The Differential Diagnosis of Some Forms of Headache."

In the discussion Dr. Geo. F. Cott spoke from the standpoint of the rhinologist, the nose being often the cause of many forms of headaches. He cited the case of one man who visited the hospital in Philadelphia with a severe headache that had existed for years. Several neurologists had made a diagnosis of hysteria, but no result from any form of treatment. His was given the rotary test and trouble was shown to be in the sinuses, but no operation was made. In a day or two this man was picked up unconscious on the street and died in the hospital, a post mortem showed the rupture of an abscess which had been located in the sinuses, had been an extension from the nose, and could have been relieved had operation been performed before.

Dr. Dowd brought out a very important point, nerve-cell starvation as a cause of neurasthenia, the latter being the cause of many headaches.

The following case was reported in which the specialist treating the eyes acknowledged, had the general neurasthenic condition been remedied by the general practitioner the patient would have been saved the trouble and expense of consulting the ophthalmologist.

Miss L. For months repeated attacks of stys, always more or less headache, there were also many symptoms of nerve-cell tire, neurasthenia present. Refraction with the fitting of proper glasses seemed not to relieve, in fact they so aggravated the condition that she could not use them at all with any ease. Nose pieces were changed but no relief, rather the whole general condition was made apparently worse. Being referred for examination as to renal condition, I found no albumin or casts, no sugar, pus or blood, a considerable increase in indican and a Phosphatic Index 80% minus.

Being inclined to hysteria Fl. Ex. Valerian M 20, Comp. Phos. Tonic M 25 was ordered in glycerine three times daily in milk half an hour after food.

The headaches ceased in about three or four days, her general condition improved, at the end of two weeks albumin had disappeared and Index was noted to be gradually raising. At the end of nine months this lady was seen again, she had gained 15 pounds in weight, had not had an attack of stys, there was no headache and she said she found it was not necessary to wear the glasses.

Dinner was served at the Inn, several invited guests being present, including Major Rucker, U. S. A., in charge of the Base Hospital at Fort Porter.

---

The Buffalo Chapter of **Nu Sigma Nu** held a smoker at the chapter house, Oct. 18.

---

**The Steuben Co. Medical Society** held its annual meeting at Bath, Oct. 17 and elected the following officers: Pres. Frank H. Swain, Corning; Vice Pres. R. C. Spaulding, Cohocton; Sec.-Treas. B. R. Wakeman, Hornell; Censors, Drs. Smith of Corning, Spaulding of Cohocton, Stewart of Canisteo, Aldrich of Addison.

